# Review of the genus *Harnischia* Kieffer from China (Diptera, Chironomidae), with description of one new species

**DOI:** 10.3897/zookeys.634.10323

**Published:** 2016-11-21

**Authors:** Chun-Cai Yan, Qin Guo, Ting Liu, Wei Guo, Xin-Hua Wang, Bao-Ping Pan

**Affiliations:** 1 Tianjin Key Laboratory of Animal and Plant Resistance, Tianjin Normal University, Tianjin, 300387, PR China; 2 College of Life Sciences, Nankai University, Tianjin 300071, China

**Keywords:** Harnischia, Chironomidae, China, new species, key

## Abstract

The genus *Harnischia* Kieffer, 1921 from China is reviewed and one new species, *Harnischia parallela* Yan & Wang, **sp. n.**, is described and illustrated as adult male. *Harnischia okilurida* Sasa is recognized as a new synonym of *Harnischia longispuria* Wang & Zheng. The pupae of *Harnischia fuscimana* Kieffer and *Harnischia curtilamellata* Malloch are redescribed from material collected in China, and an unplaced pupa is described. Key to male adults and pupae of known species of *Harnischia* from China is given.

## Introduction

The genus *Harnischia* is characterized by the morphology of the hypopygium, which is bearing a vestigial superior and inferior volsella. Previously erected by Kieffer (1921), this genus is treated as a group within Tendipes (Cryptochironomus) by Goetghebuer (1937–54). Townes (1945) proposed dividing the genus into two subgenera (*Harnischia* and *Cladopelma*). Beck and Beck (1969) also treated the genus as two subgenera. Recently, Sæther (1977) elevated *Cladopelma* to the genus level. The *Harnischia* genus has a worldwide distribution (Cranston and Martin 1989; Ashe and Cranston 1990; Chaudhuri and Chattopadhyay 1990; Oliver et al. 1990; Wang et al. 1993; Sasa and Kikuchi 1995; Sasa and Ogata 1999; Wang 1999; Sæther et al. 2000; Wang 2000; Chaudhuri et al. 2001; Kawai et al. 2002, Makarchenko et al. 2005; Yan and Wang 2011; Paasivirta 2014; Murray 2015).

Wang (1999) recorded six species from China and recognized *Harnischia longispuria*, which is erected by Wang et al. (1993) as a synonym of *Harnischia curtilamellata*. After re-examination of the type specimen, *Harnischia longispuria* has been treated as a valid species (Wang 2000, Yan and Wang 2011). To date, seven *Harnischia* species (*Harnischia angularis*, *Harnischia cultriata*, *Harnischia curtilamellata*, *Harnischia fuscimana*, *Harnischia japonica*, *Harnischia longispuria* and *Harnischia turgidula*) of the genus have been recorded in China.

In this paper the genus *Harnischia* from China is reviewed and a description of the male adult of *Harnischia parallela* Yan & Wang, sp. n., is given. *Harnischia okilurida* Sasa is recognized as a new synonym of *Harnischia longispuria* Wang & Zheng. The pupae of *Harnischia fuscimana* Kieffer and *Harnischia curtilamellata* Malloch are redescribed from China, and an unplaced pupa is described. Key to male adults and pupae of known species of *Harnischia* from China is provided.

## Material and methods

Morphology and terminology follow Sæther (1980). The material examined consists of slide-mounted following the procedures outlined by Sæther (1969). Measurements are given as the ranges followed by a mean when four or more measurements are made. The specimens examined in this study are deposited in the collection of the College of Life Sciences, Tianjin Normal University, China (BDN).

## Taxonomy

### Harnischia

Taxon classificationAnimaliaDipteraChironomidae

Kieffer, 1921

Harnischia : Kieffer 1921: 273; Beck and Beck 1969: 296; Sæther 1971: 350; 1977: 89; Pinder and Reiss 1986: 326; Sasa 1989: 83; Cranston et al. 1989: 382; Langton 1991: 275; Wang 1995: 169; Rufer and Ferrington 2007: 82.

#### Type species.

*Harnischia fuscimana* Kieffer, 1921.

#### Diagnostic characters.

***Adult male*.** The characters of superior and inferior volsellae vestigial and gonostylus broad and short, not attenuated from junction with gonocoxite will easily differentiate *Harnischia* adults from other genera in the subfamily Chironominae. ***Pupae*.** The medially interrupted hook row, pattern of armature on the tergites, absence of posterolateral comb on segment VIII and brush-like thoracic horn will differentiate the pupae from all the other chironomids.

### Harnischia angularis

Taxon classificationAnimaliaDipteraChironomidae

Albu & Botnariuc, 1966

Fig. 1

Harnischia angularis : Albu and Botnariuc 1966: 54; Wang 1999: 169; Wang 2000: 644; Makarchenko et al. 2005: 410.
Harnischia hamata : Wang et al. 1993: 461; Wang 1999: 169. **Syn. n.**

#### Type locality.

Romania.

#### Material examined.

China: 1♂, Yunnan Province, Huaping Country, Xinzhuang Town, Liangma River, 30.05.1996, sweep net, X. Wang; 1♂, Ningxia, Yinchuan City, 26.07.1987, Wang; 2♂♂, Xinjiang, Buerjin Hotel, 1.09.2002, light trap, H. Tang.

#### Diagnostic characters.

Frontal tubercles absent. R_1_ without microtrichia. Tibia of front leg with a subapical seta; posterior margin of tergite IX narrowed, anal point elongated, swollen medially, sharp and slender, without lateral setae. Anal tergite bands V-shaped, abruptly interrupted medially; gonocoxite with a projection in inner distal parts, bearing 5 strong setae and covered with microtrichia. Gonostylus slightly swollen distally, with hook-like apical teeth at the apex.

**Figure 1.**
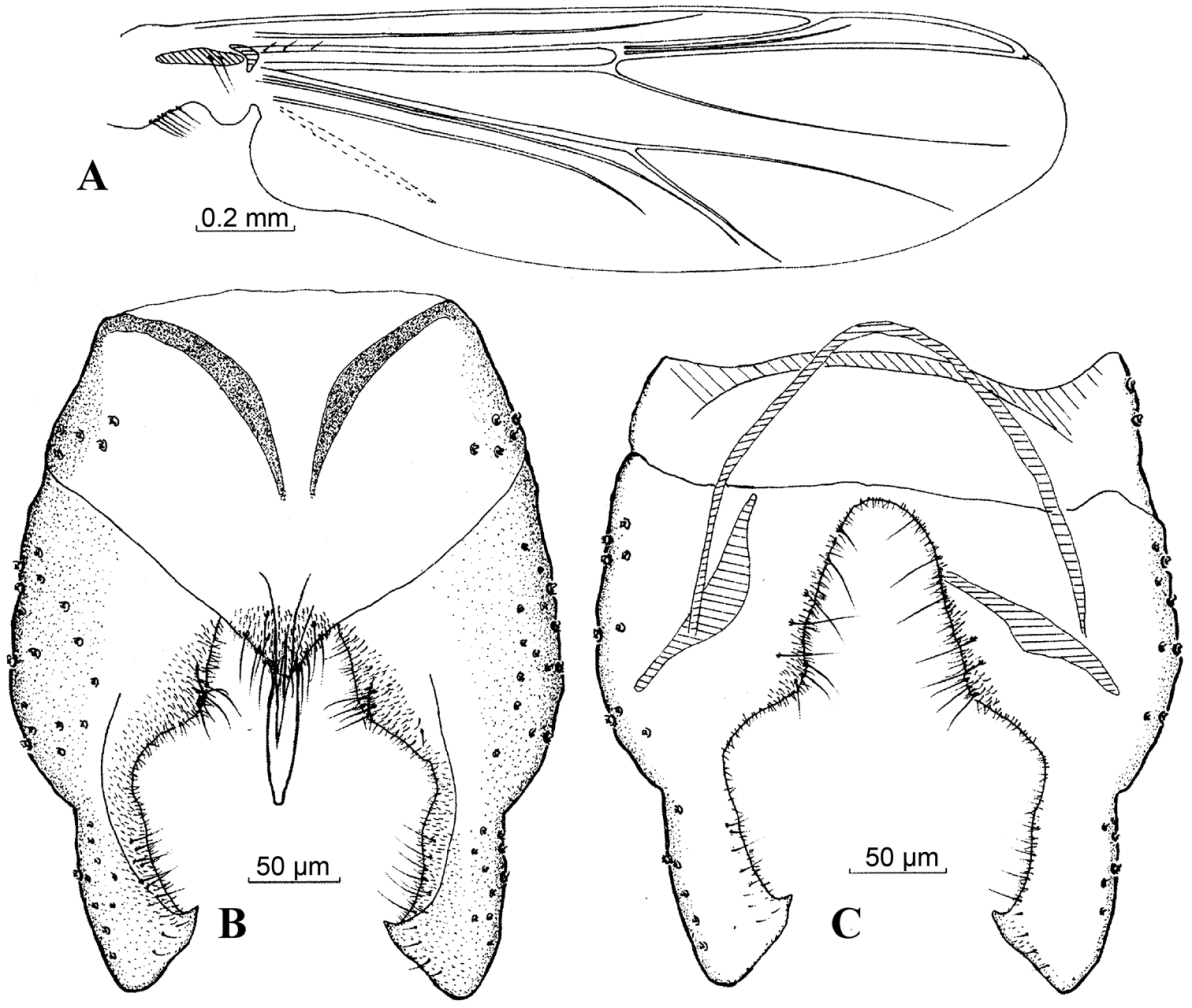
*Harnischia angularis*. **A** Wing; Hypopygium: **B** (dorsal) **C** (ventral).

#### Distribution.

China (Yunnan, Ningxia, Xinjiang); Russian Far-East; Germany, Yugoslavia, Romania, Italy.

#### Remarks.

The species can be easily distinguished by gonostylus with strong hook-like apical teeth. We agree with Wang (1999), who considered *Harnischia hamata* Wang & Zheng as a synonym of *Harnischia angularis* Albu & Botnariuc.

### Harnischia cultriata

Taxon classificationAnimaliaDipteraChironomidae

Wang, 1999

Fig. 2

Harnischia cultriata Wang 1999: 172.

#### Type locality.

China (Gansu).

#### Material examined.

***Holotype***: ♂, (BDN no. 03438), China: Gansu Province, Tianshui City, Dangchuan Country, 1450 m, 8. viii. 1993, sweeping net. W. Bu.

#### Diagnostic characters.

Frontal tubercles absent. R_1_ without microtrichia. Tergite IX with shoulder-like posterior margin. Anal point slightly swollen in the distal 1/3, sharp at the apex, with median ridges but without lateral setae. Anal tergite bands Y-shaped, slightly flat at the bottom. Superior volsella present, bearing 4 strong setae and microtrichia. Gonocoxite with an obvious projection in distal portion of inner margin, bearing 2–3 strong setae and microtrichia; Gonostylus swollen- to knife-like at the middle, with sharp teeth at the apex.

**Figure 2.**
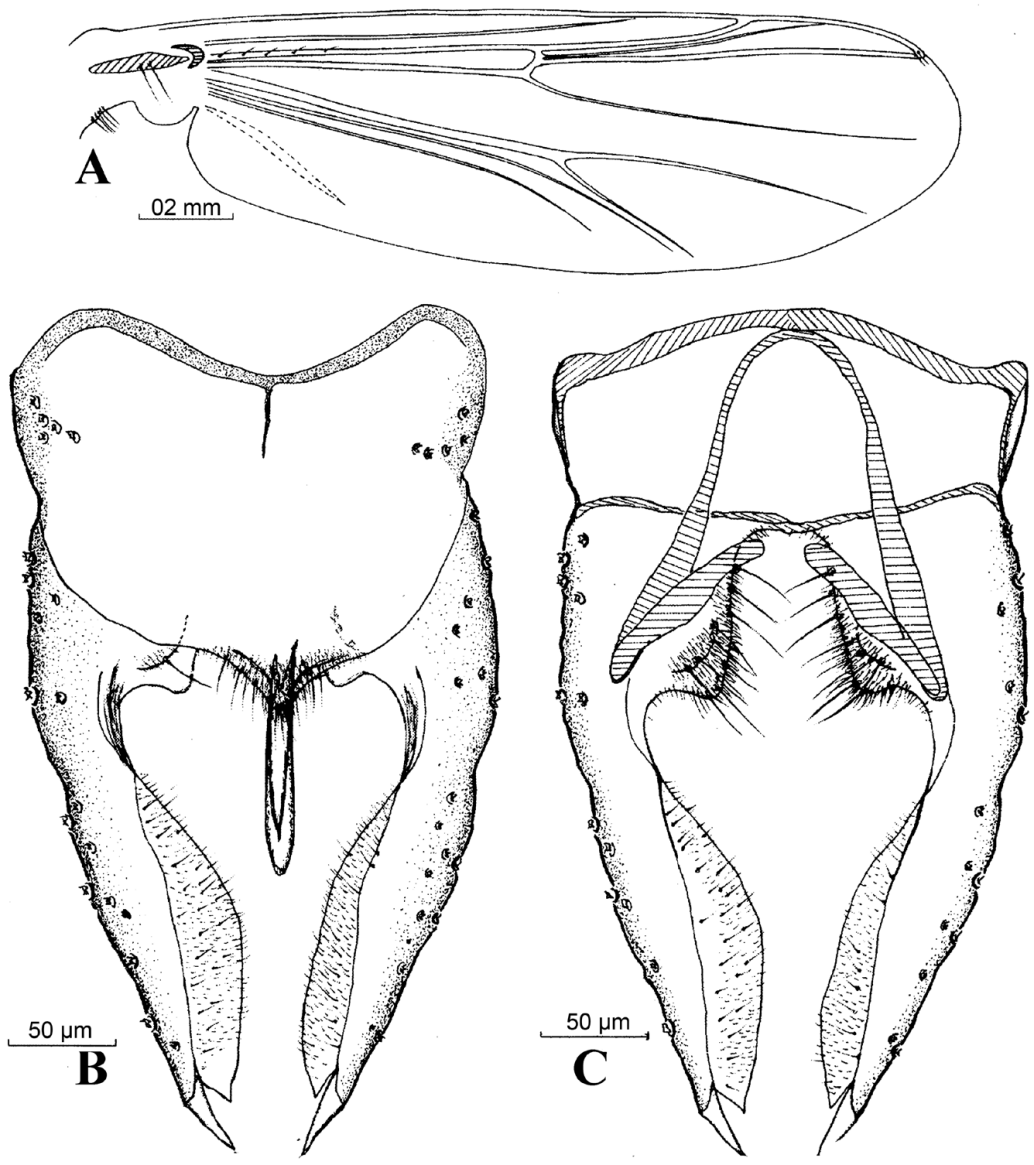
*Harnischia cultriata*. **A** Wing; Hypopygium: **B** (dorsal) **C** (ventral).

#### Distribution.

China (Gansu).

### Harnischia curtilamellata

Taxon classificationAnimaliaDipteraChironomidae

(Malloch, 1915)

Figs 3 , 4

Chironomus curtilamellata Malloch 1915: 474.
Chironomus pseudosimplex Goetghebuer 1923: 116; Edwards 1929: 390.
Chironomus (Cryptochironomus) monilis Freeman 1954a: 19.
Chironomus (Cryptochironomus) atrofasciatus Freeman 1954b: 177.
Harnischia pseudosimplex Wang S. et al. 1977: 231.
Harnischia curtilamellata : Townes 1945: 166; Sæther 1971: 347; 1977a: 88; Pinder 1978: 124; Freeman and Cranston 1980: 351; Hashimoto et al. 1981: 22; Sasa and Kikuchi 1986: 20; Sasa and Kawai 1987: 18; Ashe and Cranston 1990: 285; Cranston and Martin 1989: 270; Wang X. et al. 1993: 462; Makarchenko et al. 2005: 410; Dutta et al. 1996: 272; Wang 1999: 170; Wang 2000: 644; Chaudhuri et al. 2001: 351.

#### Type locality.

America (Michigan).

#### Material examined.

China: 3♂♂, Tianjin City, Xian River, 12.06.1985, Xinhua Wang; 1 ♂, Jiangxi Province, Poyang Lake, Nanjishan Natural Conservation area, 12.06.2004, sweep net, Chuncai Yan. 2 ♂♂, Hubei Province, Hefeng Watershed, 1200 m, 17.07.1999, light trap, Bingchun Ji; 1 ♂, Hunan Province, Zhuzhou City, Central South Forestry University, 17.07.1995, Winjun Bu; 1 ♂, Guangxi Province, Jinxiu County, 1.06.1990, Xinhua Wang; 1 ♂, Hainan Province, Ledong Li Autonomous County, Jianfengling, 17.04.1985, light trap, Leyi Zheng; 2 ♂♂, Guizhou Province, Guiyang City, Huaxi area, 1050 m, 23.07.1995, light trap,Wenjun Bu; 1 ♂, Guizhou Province, Daozhen County, Dashahe Natural Conservation area, Fairy Cave, 600 m, 30.05.2004, light trap, Hongqu Tang; 1 ♂, Guizhou Province, Jiangkou County, Fanjingshan Natural Conservation area, Black Creek, 3.06.2002, light trap, Bingchun Ji; 9 ♂♂, Yunnan Province, Dalier Seaside, 2000 m, 23.05.1996, light trap, Xinhua Wang; 3 ♂♂, Yunnan Province, Eryuan County, Niujie Town, Futian, Meici River, 2262–2332 m, 14.5°C, 24.05.1996, light trap, Changfa Zhou and Beixin Wang; 1 ♂, Yunnan Province, Lijiang City, Shigu Town, Chongjiang River, 25.05.1996, light trap, Changfa Zhou; 1 ♂, Yunnan Province, Wuding County, Mashan Stream, 1.06.1996, Xinhua Wang; 1 ♂, Yunnan Province, Zhongdian Martyr Cemetery, 3150 m, 14.07.2001, Ruilei Zhang; 28 ♂♂, Taiwan, Taibei City, Kuandu Wetland, 20.10.1988, Kanjin Ma.

#### Diagnostic characters.

**Figure 3.**
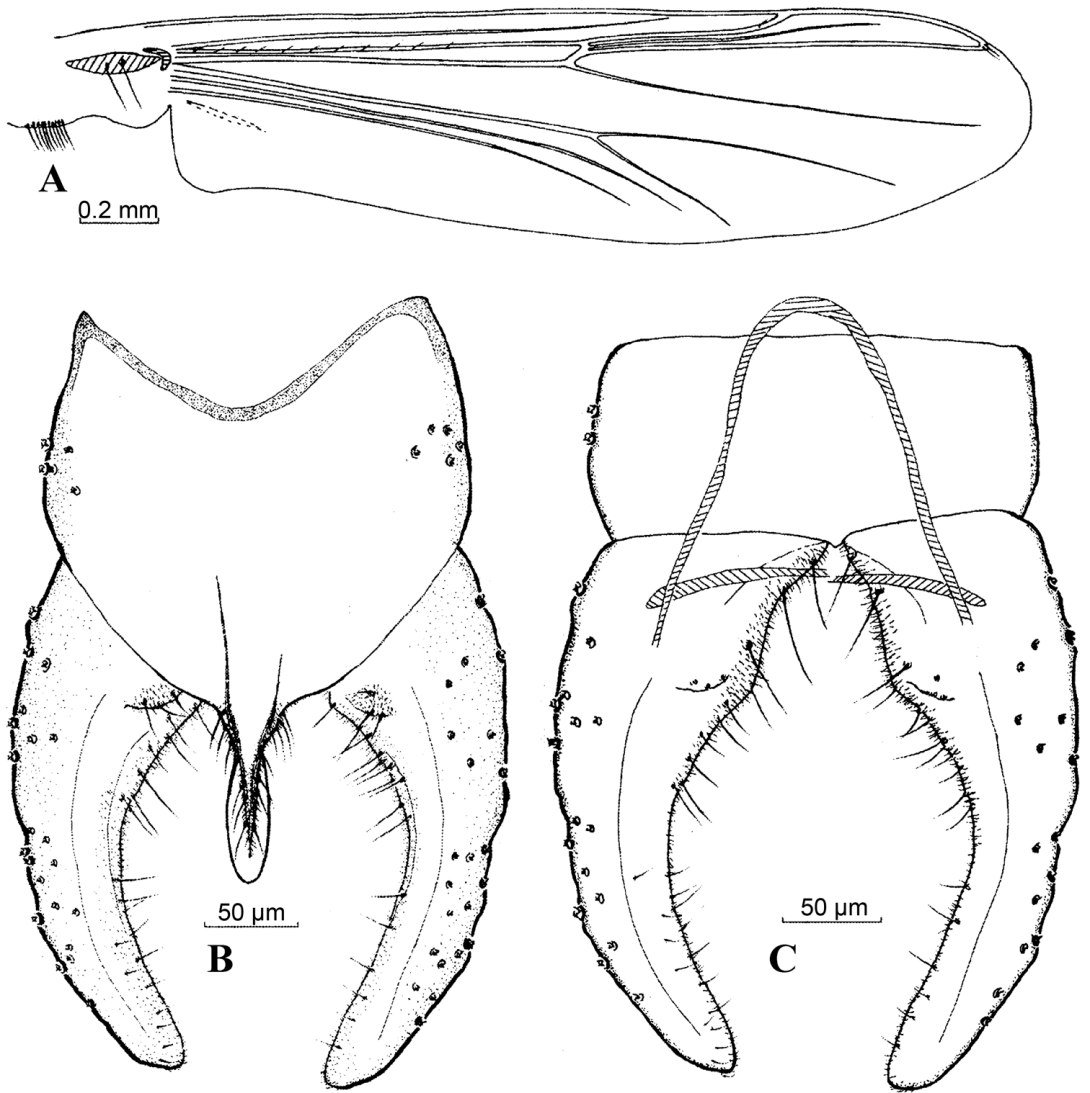
*Harnischia curtilamellata*. **A** Wing; Hypopygium: **B** (dorsal) **C** (ventral).

**Figures 4.**
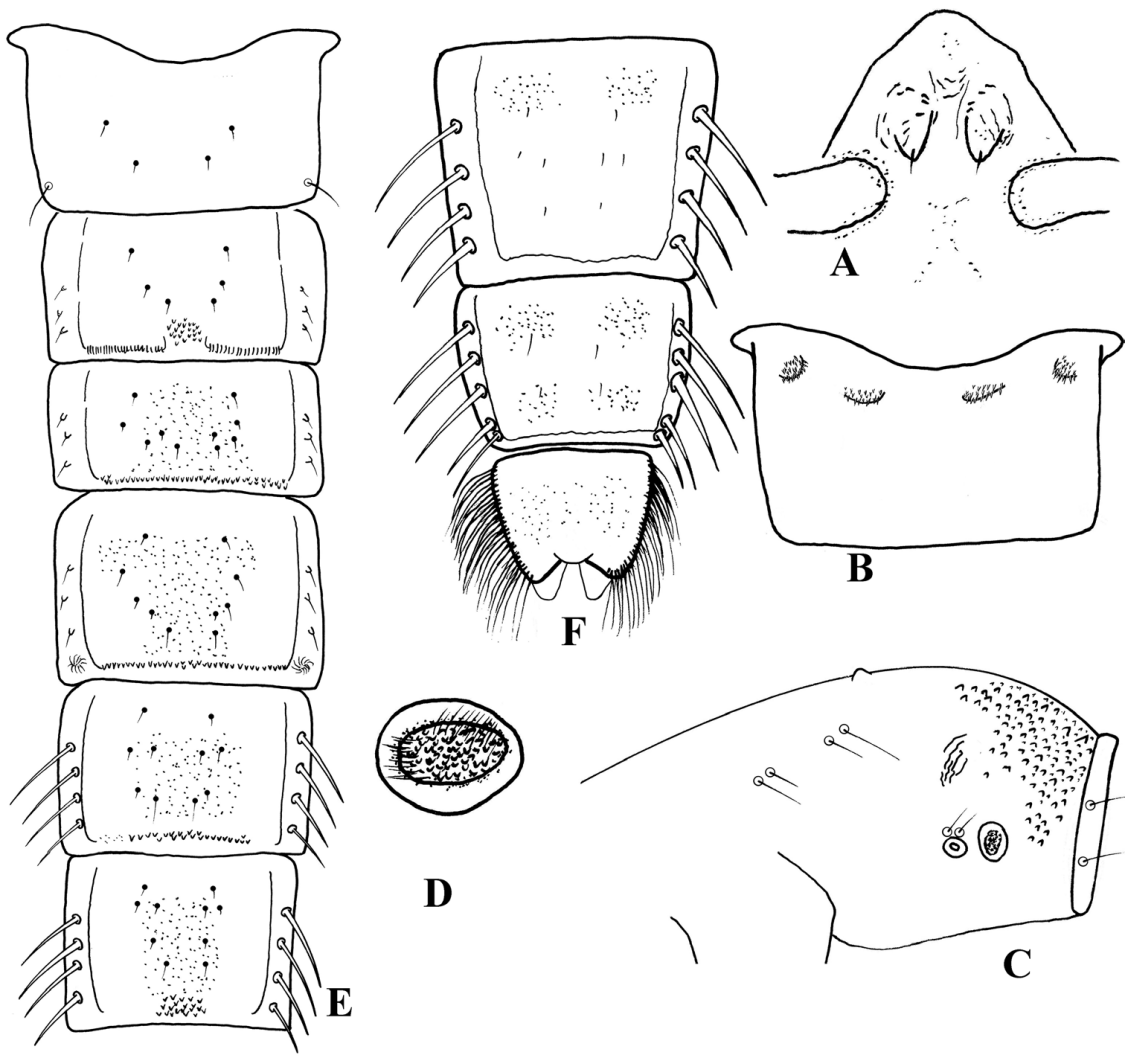
*Harnischia curtilamellata*. Pupae. **A** frontal apotome **B** sternite I **C** thorax, lateral view **D** basal ring **E** tergites I–VI **F** tergite VII–VIII and anal lobe.

***Male*.** (Fig. 3A–C) Frontal tubercles elliptical or rounded, sometimes absent. Anal point wide, shrunken and slender at the base, distally swollen like a bubble, semi-transparent, with median ridges, bearing lateral setae and microtrichia. Phallapodeme long and slender. Gonostylus widest at the base, curved in the middle and gradually tapering distally, round and blunt in the apex. ***Pupa*.** (Fig. 4A–F) Sternite I with a pair of spinose anterolateral and anteromedian tubercles on each side; tergites III-VI with narrow, posterior robust spines; hook row medially interrupted; segment VIII without comb or spurs.

#### Materials examined.

China: 1P, Guangxi Zhuang Autonomous Region, Guilin City, Dingjiang County, Yangjiangtou town 6.04.2015. Wenbin Liu. 1P, China: Shandong Province, Jining City, Weishan County, Weishan Island 4.10.2015. Wenbin Liu. 5P, China: Guangdong Province, Maoming City, Linchen River 27.11.2013. Jun Liu.

#### Description.

***Pupa*** [n = 8] Total length 3.2–4.9 mm. Cephalothorax brown; abdomen pale brownish.

*Cephalothorax* (Fig. 4A, C–D). Frontal setae 20–30 µm long, fine, arising subapically from conical, 50–100 µm long cephalic tubercles. Thoracic horn plumose, with numerous fine branches; basal ring oval. Thorax granulose dorsally, more densely granulose anteriorly. Prealar tubercle low, rounded. Scutal tubercle prominent. Wing sheath without nose; pearl row absent. Two short precorneals, 2 short antepronotals and 4 dorsocentrals present. Lengths of dorsocentrals (µm): 50–75, 38–50, 56–63, 25–40.

*Abdomen* (Fig. 4B, E–F). Tergite I (Fig. 4E) bare; II with posteromedian group of small points; III-VI with narrow, transverse, posterior band of robust spines; VII with a pair of anterior patches fine shagreen; VIII with a pair of anterior and posterolateral patches of shagreen. Hook row widely interrupted medially. Conjunctives bare. Pedes spurii A present on segment IV; pedes spurii B present on segment I. Sternite I (Fig. 4B) with a pair of spinose anterolateral and anteromedian tubercles each sides. Segment VIII without posterolateral comb or spurs. Segment II-IV with 3 strong L setae situated on tubercles; V-VII with 4 LS setae, VIII with 5 LS setae.

*Anal lobe* (Fig. 4F) 1.44–2.20 × as long as broad, with complete fringe of 31–51 lamelliform setae. Genital sac 125–220 µm long, extending beyond anal lobe.

#### Distribution.

China (Tianjin, Jiangxi, Hubei, Hunan, Guangxi, Hainan, Guizhou, Yunnan, Taiwan); Japan; Thailand; India; Russian Far–East; Europe; North America; Africa region (South Africa, Sudan, Sebegal, Zaire); Australian Region (Australia).

#### Remarks.

The Chinese specimens mainly agree with the description of Langton (1991), but the color of spinose anteromedian tubercles of sternite I is lighter than from Europe. Based on original descriptions and figures, the record of *Harnischia pseudosimplex* Goetghebuer in China (Hubei province; Wuhan City) by Wang S. et al. (1977: 231, fig. II: 15) should be *Harnischia curtilamellata* (Malloch).

### Harnischia fuscimana

Taxon classificationAnimaliaDipteraChironomidae

Kieffer, 1921

Fig. 5

Harnischia fuscimana : Kieffer 1921b: 69; Goetghebuer 1937–54: 48; Sæther 1971: 348; Wang S. et al. 1977: 231; Fittkau and Reiss 1978: 432; Wang 2000: 644; Makarchenko et al. 2005: 410.

#### Type locality.

Poland

#### Diagnostic characters.

**Figures 5.**
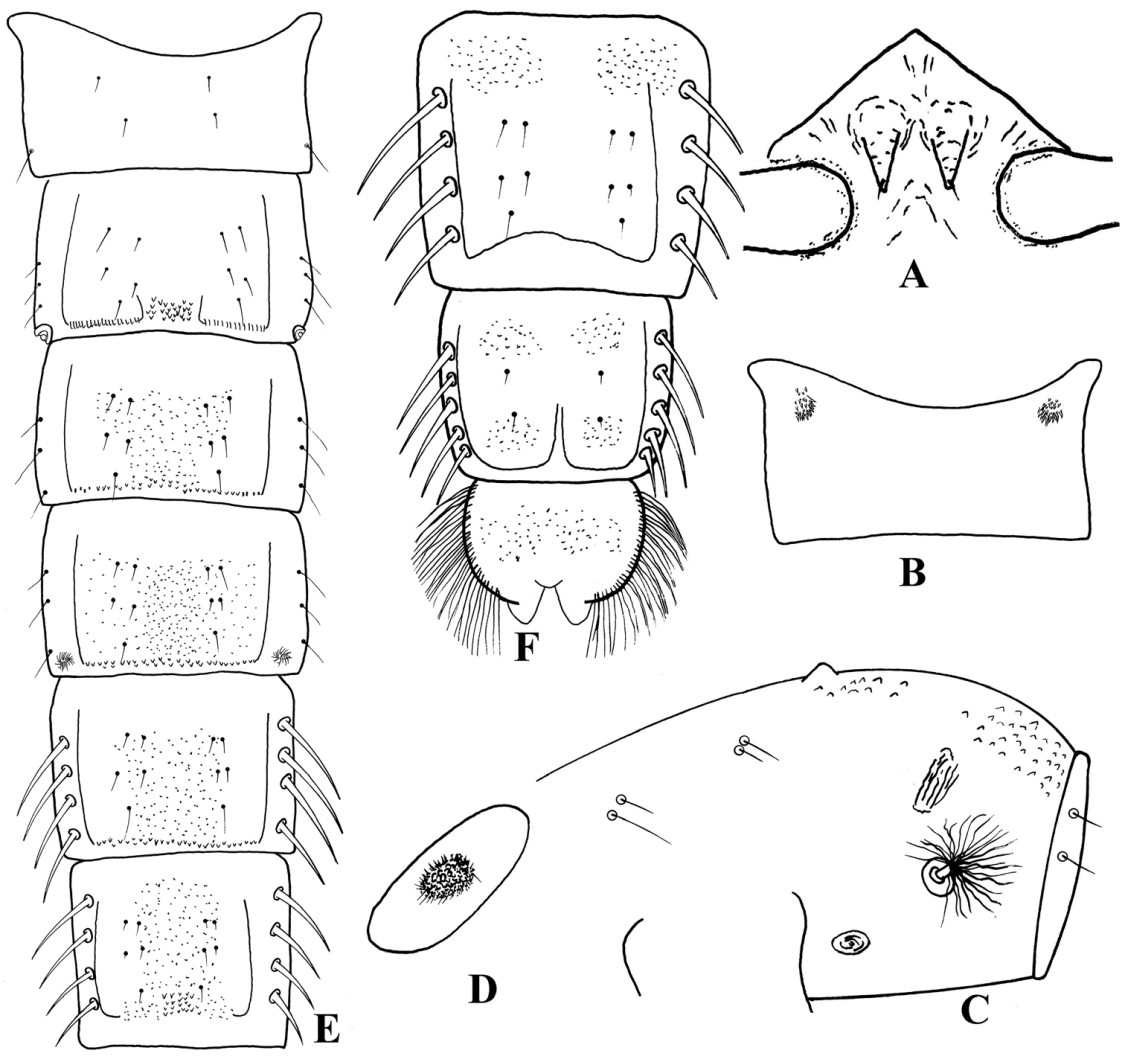
*Harnischia fuscimana*. Pupae. **A** frontal apotome **B** sternite I **C** thorax, lateral view **D** basal ring **E** tergites I–VI **F** tergite VII–VIII and anal lobe.

***Male*.** Body pale green. Anal point taper-shaped and tapering towards the sharp apex, bearing lateral setae and microtrichia. Gonostylus thick, blade-shaped projection. Superior and inferior volsellae absent. The conjunction of gonostylus and gonocoxite shrunken and obviously tapered. Gonostylus short, with a weak projection at base, almost as long as gonocoxite. ***Pupa*.** (Fig. 5A–F) Sternite I with only spinose anterolateral tubercles; hook row medially interrupted; segment VIII without comb or spur.

#### Materials examined.

China: 1P, Guangdong Province, Maoming City, Baowei town 10.12.2012. Hongqu Tang.

#### Description.

***Pupa*** [n = 1] Total length 6.55 mm. Cephalothorax brown; abdomen pale brownish.

*Cephalothorax* (Fig. 5A, C–D). Frontal setae 45 µm long, fine, arising subapically from conical, 130 µm long cephalic tubercles. Thoracic horn (Fig. 5C) plumose, with numerous fine branches; basal ring oval. Thorax granulose dorsally, more densely granulose anteriorly. Prealar tubercle low, rounded. Scutal tubercle prominent. Wing sheath without nose; pearl row absent. Two short precorneals, 2 short antepronotals and 4 dorsocentrals present.

*Abdomen* (Fig. 5B, E–F). Tergite I (Fig. 5E) bare; II with posteromedian group of small points; III-VI with transverse, posterior band of spinules; VII with a pair of anterior patches fine shagreen; VIII with a pair of anterior and posterolateral patches of shagreen. Hook row widely interrupted medially. Conjunctives bare. Pedes spurii A present on segment IV; pedes spurii B present on segment I and II. Sternite I (Fig. 5B) with spinose anterolateral tubercles. Segment VIII without posterolateral comb or spur. Segment II-IV with 3 strong L setae; V-VII with 4 LS setae, VIII with 5 LS setae.

*Anal lobe* (Fig. 5F) 1.74 × as long as broad, with complete fringe of 70 lamelliform setae. Genital sac 300 µm long, extending beyond anal lobe.

#### Distribution.

China (Hubei), Russian Far East; Afghanistan; Lebanon; Europe (Gemary, Poland, Yugosiavia, Romania, France, Belgium, Spain, Italy).

#### Remarks.

The species was recorded in China by Wang S. (1977). The Chinese specimens of pupal stages mainly agree with the description of Langton (1991), but fringe setae of anal lobe of specimens from China (70) more than from Europe (45–59).

### Harnischia japonica

Taxon classificationAnimaliaDipteraChironomidae

Hashimoto, 1984

Fig. 6

Harnischia japonica : Hashimoto 1984: 262; Wang et al. 1993: 461; Sasa et al. 1988: 32; Sasa 1990a: 31; Sasa 1993: 72; Wang 1999: 172; Wang 2000: 644; Makarchenko et al. 2005: 410.

#### Type locality.

Japan.

#### Material examined.

China: 1 ♂, Fujian Province, Wuyi Mountain Natural Conservation area, 24.04.1993, light trap, Xinhua Wang; 3 ♂♂, Shandong Province, Yantai City, Mouping, Kunyu Mountain, Dianhou, 24.08.1987, Hongyang Li; 2 ♂♂, Guangxi Province, Longsheng County, Sanmen Town, 27.05.1990, Xinhua Wang. Korea: 1 ♂, 20.05.2000, Dr. T.S. Chon.

#### Diagnostic characters.

Ventral tergites I-IV each terminal with brown band. R_1_ without microtrichia. Tergite IX with shoulder-like posterior margin. Anal point slightly swollen in the distal 1/3, round and blunt at the apical and with median ridges, bearing lateral setae and microtrichia, stretching to the middle of tergite IX. Anal Tergite bands Y-shaped. Inner margin of gonocoxite with a small protrusion in distal, bearing setae and microtrichia. Gonostylus slender at the base, swollen and truncated apically.

**Figures 6.**
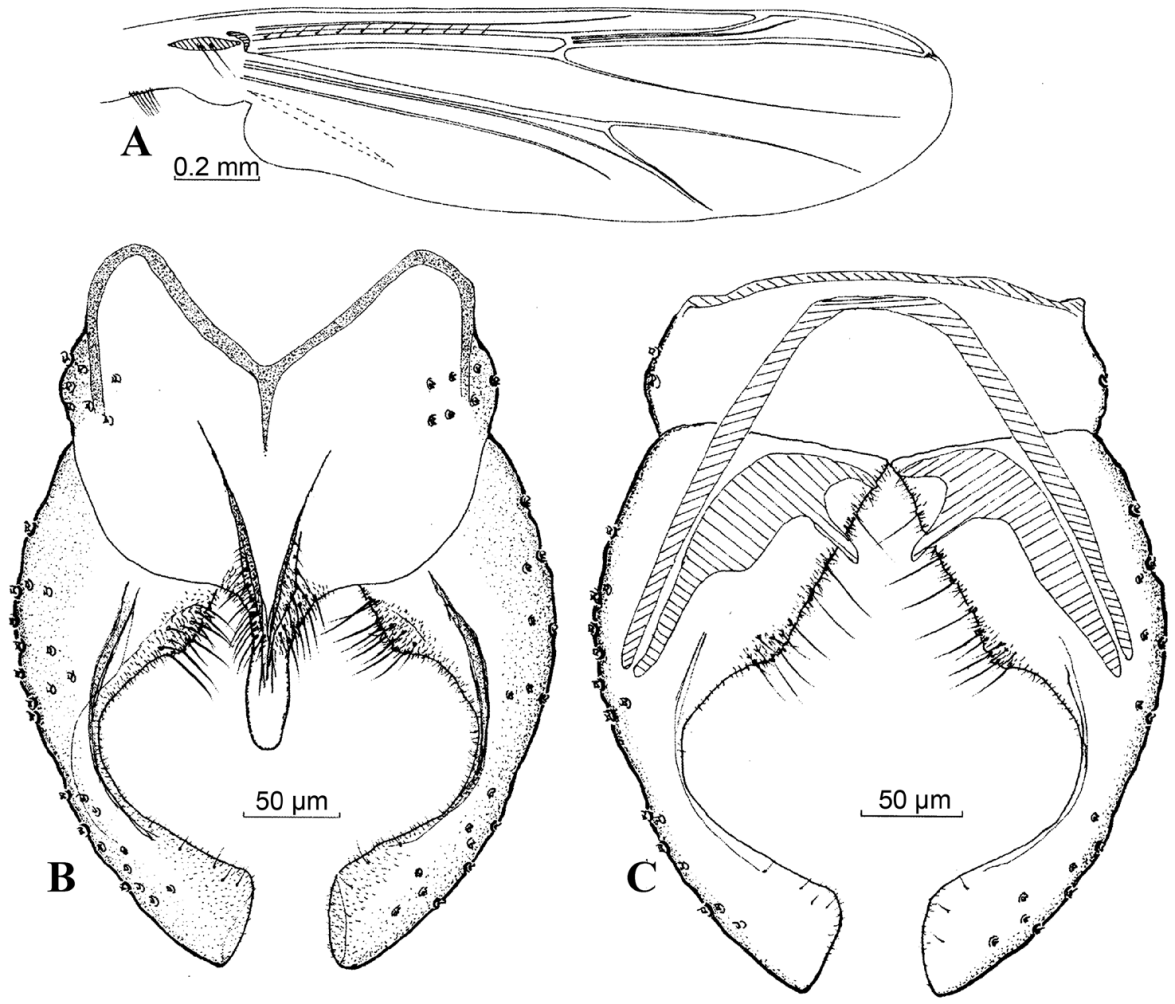
*Harnischia japonica*. **A** Wing; Hypopygium: **B** (dorsal) **C** (ventral).

#### Distribution.

China (Fujian, Shandong, Guangxi); Korea; Japan; Russian Far East.

### Harnischia longispuria

Taxon classificationAnimaliaDipteraChironomidae

Wang & Zheng, 1993

Fig. 7

Harnischia longispuria : Wang and Zheng 1993: 459; Wang 1999: 170; Wang 2000: 644.
Harnischia okilurida : Sasa 1993: 127 **Syn. n.**

#### Type locality.

China (Hainan).

#### Material examined.

China: Holotype, ♂ (BDN No. 05224), Hainan Province, Ledong Li Autonomous County, Jianfeng town, 17.05.1988, light trap, Leyi Zheng.Japan: Holotype (*Harnischia okilurida* Sasa), ♂ (No. 246: 10), at the side of a dam of Yona River, Lake Nawagaike, 20.05.1993, insect net.

#### Diagnostic characters.

Thorax yellow with dark brown spots; AR 0.98, frontal tubercles absent; postrior margin of tergite IX triangular and cone-like; anal point constricted at base and swollen distally, with median ridges, bearing setae and microtrichia; anal tergite bands V-shaped, slightly flat at bottom; a fusion of gonostylus and gonocoxite obviously constricted, gonostylus straight, rod-like, both sides almost parallel, round and blunt at apex, without conspicuous short setae in inner margin.

**Figures 7.**
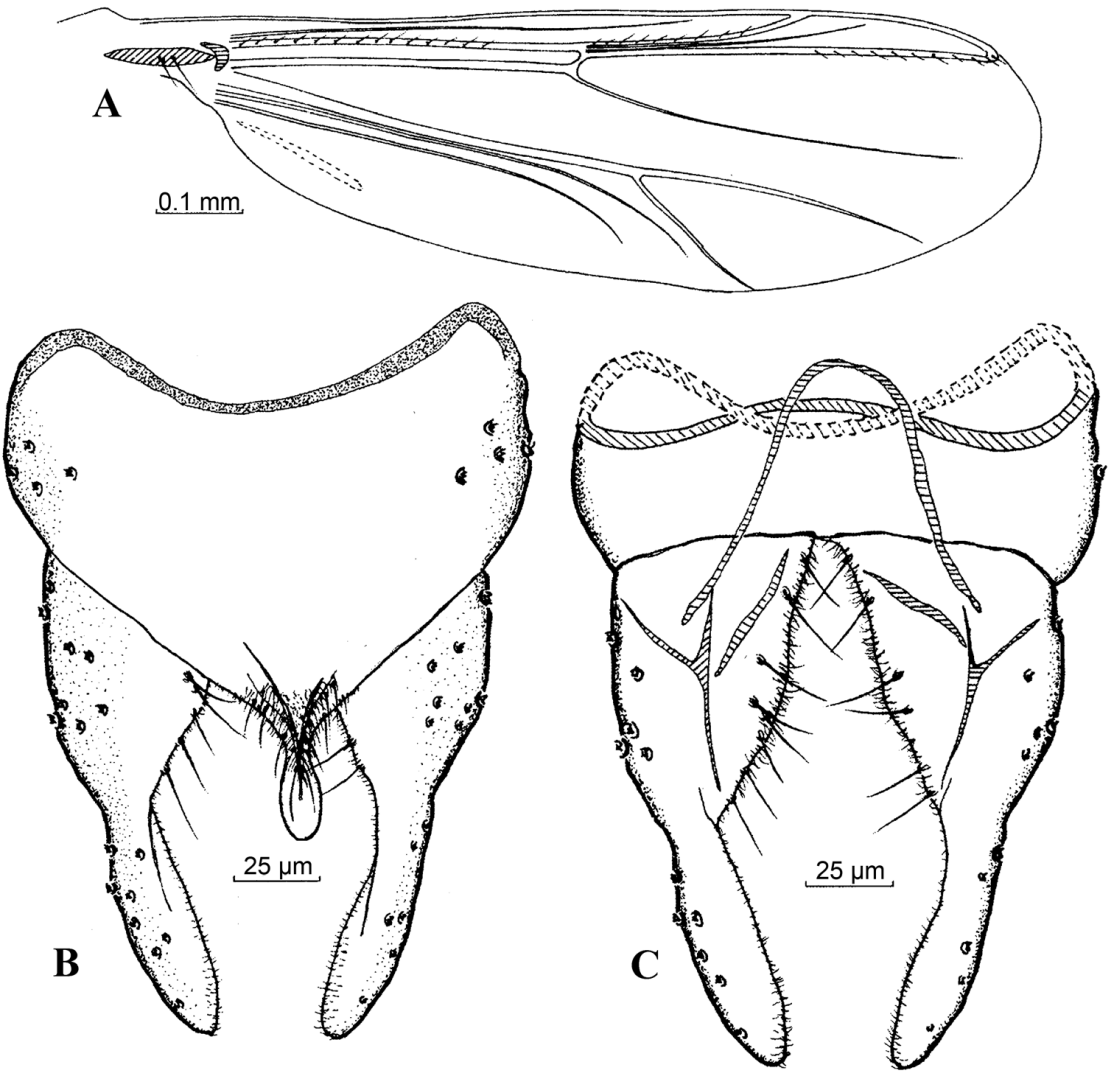
*Harnischia longispuria*. **A** Wing; Hypopygium: **B** (dorsal) **C** (ventral).

#### Distribution.

China (Hainan); Japan.

#### Remarks.

Wang (1999) treat *Harnischia longispuria* Wang & Zheng as a synonym of *Harnischia curtilamellata* (Malloch). After re-examining, the specimens of *Harnischia longispuria* Wang & Zheng, (which should be *Harnischia okilurida* Sasa), it currently should be a valid species. However, *Harnischia okilurida* Sasa (December 1993) described later than *Harnischia longispuria* Wang & Zheng (October 1993), is consequently considered as a new synonym of *Harnischia longispuria* Wang & Zheng.

### Harnischia parallela

Taxon classificationAnimaliaDipteraChironomidae

Yan & Wang sp. n.

http://zoobank.org/CC353CD1-D115-4F2D-85BD-19446E7C2B00

Fig. 8

#### Diagnostic characters.

The new species is distinguished by the following combination of characters: R_1_ without microtrichia; front tibia with a subapical seta; tergite IX broadly triangular, with 26 setae (13 on each side); anal point parallel-sided, distinctly extended basally as a V-shaped form; without lateral and dorsal setae; phallapodeme wide and large; gonocoxite blunt and rounded apically, with 2 broad dorsal lobes; gonostylus with a small dorsal basal lobe bearing setae.

#### Type material.

**Holotypes**: ♂ (BDN No. 24949), China: Xinjiang burqin hotel (49.41°N, 86.59°E), 1.09.2002, Light trap, H. Tang; Paratype: 3 ♂♂ (BDN No. 24900, 24915, 24968), as holotypes.

#### Etymology.

The specific name, from Latin *parallela*, refers to anal point parallel-sided.

#### Description.

***Male imago*** [n = 4, unless otherwise stated] Total length 3.55–3.68, 3.63 mm; wing length 1.83–1.98, 1.93 mm; total length / wing length 1.86–1.94, 1.88; wing length /length of profemur 2.38–2.54, 2.47.

*Coloration*. Thorax yellow brown, with dark brown spots. Femora of front legs yellow green, tibia dark brown, tarsus 1 dark brown except for yellow brown in basal 1/2 yellow brown, tarsi 2–4 dark brown; femora and tibiae of mid and hind legs yellow green, tarsi 1–4 yellow brown to dark brown, tarsi 5 black brown. Abdomen. Tergites I–IV yellow brown, each terminal with light brown, tergites V–VIII; hypopygium dark brown.

*Head*. AR: 2.06–2.24, 2.17. Ultimate flagellomere 660–740, 710 mm. Frontal tubercles absent. Temporal setae 12–16, 14, including 3–4, 3 inner verticals; 4–5, 5 outer verticals; and 5–7, 6 postorbitals. Clypeus with 13–16, 15 setae. Tentorium 100–130, 117 mm long, 28–33, 31 mm wide. Palpomere lengths (µm): 40–45, 43(3); 42–50, 47 (3); 122–150, 137(3); 163–170, 165(3); 235–245(2); palp segment 5^th^ / 3^rd^: 1.57–1.69 (2).

*Thorax*. Antepronotum with 2–5, 4 setae, acrostichals 7–7, 7, dorsocentrals 9–11, 10, prealars 4–4, 4. Scutellum with 6–9, 7 setae.

**Figures 8.**
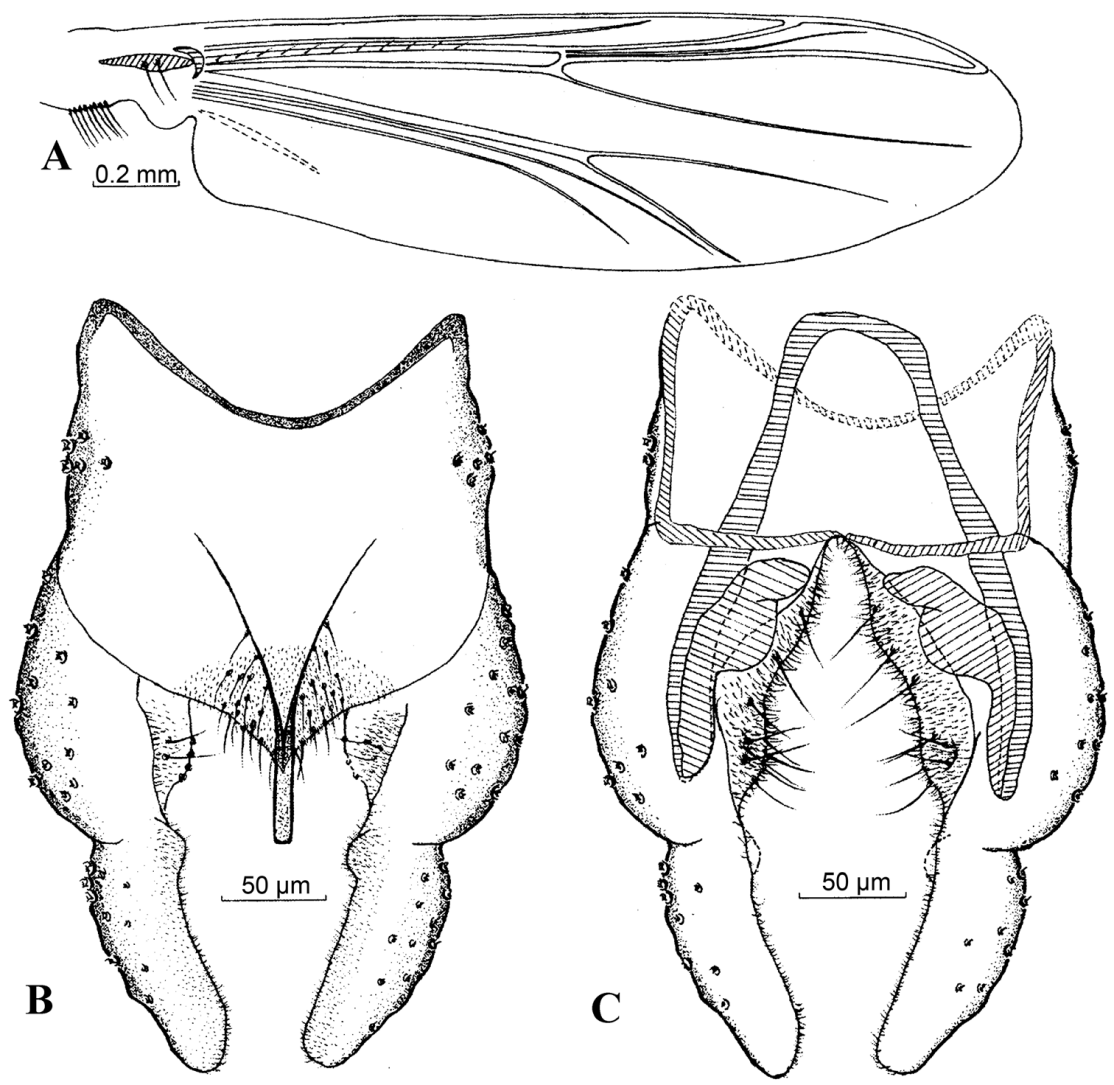
*Harnischia parallela* Yan & Wang, sp. n. **A** Wing; Hypopygium: **B** (dorsal) **C** (ventral).

*Wing* (Fig. 8A). VR: 1.11–1.13, 1.12. R with 8–11, 10 setae. R_1_ without setae. R_4+5_ with 1–2, 1 seta. Brachiolum with 2–2, 2 setae. Squama with 7–11, 9 fringed setae.

*Legs*. Front tibia with a subapical seta, 82–90, 87 mm. Mid legs with 2 spur, 15–20, 18 mm and 22–28, 26 mm, comb with 20–28, 25 teeth, 8–10, 9 mm long. Spurs of hind tibia 18–22, 20 mm and 25–32, 29 mm long, comb with 50–60, 54 teeth, 9–10, 10 mm long. Tarsus I of mid leg with 2–4, 3 sensilla chaetica. Lengths (in µm) and proportions of thoracic legs as in Table 1.

**Table 1.** Lengths (in µm) and proportions of adult male legs in *Harnischia parallela* Yan & Wang, sp. n. (n=4).

	fe	ti	ta_1_	ta_2_	ta_3_	ta_4_	ta_5_	LR
**p_1_**	730–820, 783	440–480, 460	1000–1020 1010 (3)	530–550, 540 (3)	370–390, 383 (3)	270–270, 270 (3)	130–150, 140 (3)	2.13–2.27, 2.18 (3)
**p_2_**	700–770, 745	580–650, 625	390–430, 413	190–200, 198	140–160, 150	100–110, 103	80–90, 85	0.63–0.67, 0.66
**p_3_**	800–900, 858	760–830, 810	580–640, 617 (3)	300–320, 313 (3)	260–280, 273 (3)	150–170, 160 (3)	110–110, 110 (3)	0.76–0.77, 0.77 (3)

*Hypopygium* (Fig. 8B–C). Tergite IX broadly triangular at base, bearing 26–32, 29 setae. Laterosternite IX with 4–6, 5 setae. Anal point 58–70, 65 mm long, parallel-sided, without dorsal and lateral setae. The basal ridge of anal point stretched towards the middle of tergite IX. Anal tergite bands transversally extended and concave medially. Phallapodeme 108–120, 116 mm long. Transverse sternapodeme 35–60, 47 mm long. Gonocoxite 130–147, 138 mm long, with two broad dorsal lobes, one basal and one distal, distal lobe with 8 strong setae; basal inner margin with 4 stout setae; gonostylus 117–120,119 mm long, blunt and rounded apically, with a distinct basal inner protrusion, rows of setae along inner margin absent. HR: 1.12–1.24, 1.18; HV: 3.03–3.12, 3.05.

#### Distribution.

China (Xinjiang).

#### Remarks.

*Harnischia parallela* Yan & Wang, sp. n. can be easily be separated from all other members of the *Harnischia* genus the morphological characters of: anal tergite band (transversally concave medially); anal point (parallel-sided); gonocoxite (bearing two broad dorsal lobes: 1 basal and 1 distal); gonostylus (with a small dorsal lobe at base).

### Harnischia turgidula

Taxon classificationAnimaliaDipteraChironomidae

Wang & Zheng, 1993

Fig. 9

Harnischia turgidula : Wang and Zheng 1993: 460; Wang 1999: 172; Wang 2000: 644; Makarchenko et al. 2005: 410.

#### Type locality.

China (Guangdong).

#### Material examined.

China: 4 ♂♂, Hunan Province, Yizhang County, Mang Mountain Natural Conservation area, Elevation 1200 m, 22.07.2004, Light trap, C. Yan; 4 ♂♂, Hunan Province, Yizhang County, Mang Mountain Natural Conservation area, Elevation 1280 m, 22.07.2004, Sweep net, Yan; 1 ♂, Guangdong Province, Fengkai County, Heishiding Mountain Natural Conservation area, 20.04.1988, Sweep net, Wang; 1 ♂, Guangxi Province, Longsheng, 26.05.1990, Wang; 1 ♂, Yunnan Province, Dali City, Yinqiao Town, Elevation 2000 m, 21.05.1996, Water net, Wang.

#### Diagnostic characters.

AR 1.73–2.09. Frontal tubercles absent or small. R_1_ without microtrichia. Front tibia with a subapical long seta; tergite IX shoulder-like at the posterior margin. Anal point constricted in the middle and swollen at the apex, with median ridges; anal tergite bands “V” shaped, no fusion in the middle; gonocoxite with a degenerated and small projection in inner distal, bearing setae and microtrichia; gonostylus with a swelling bubble-like protrusion at base and carry microtrichia.

**Figures 9.**
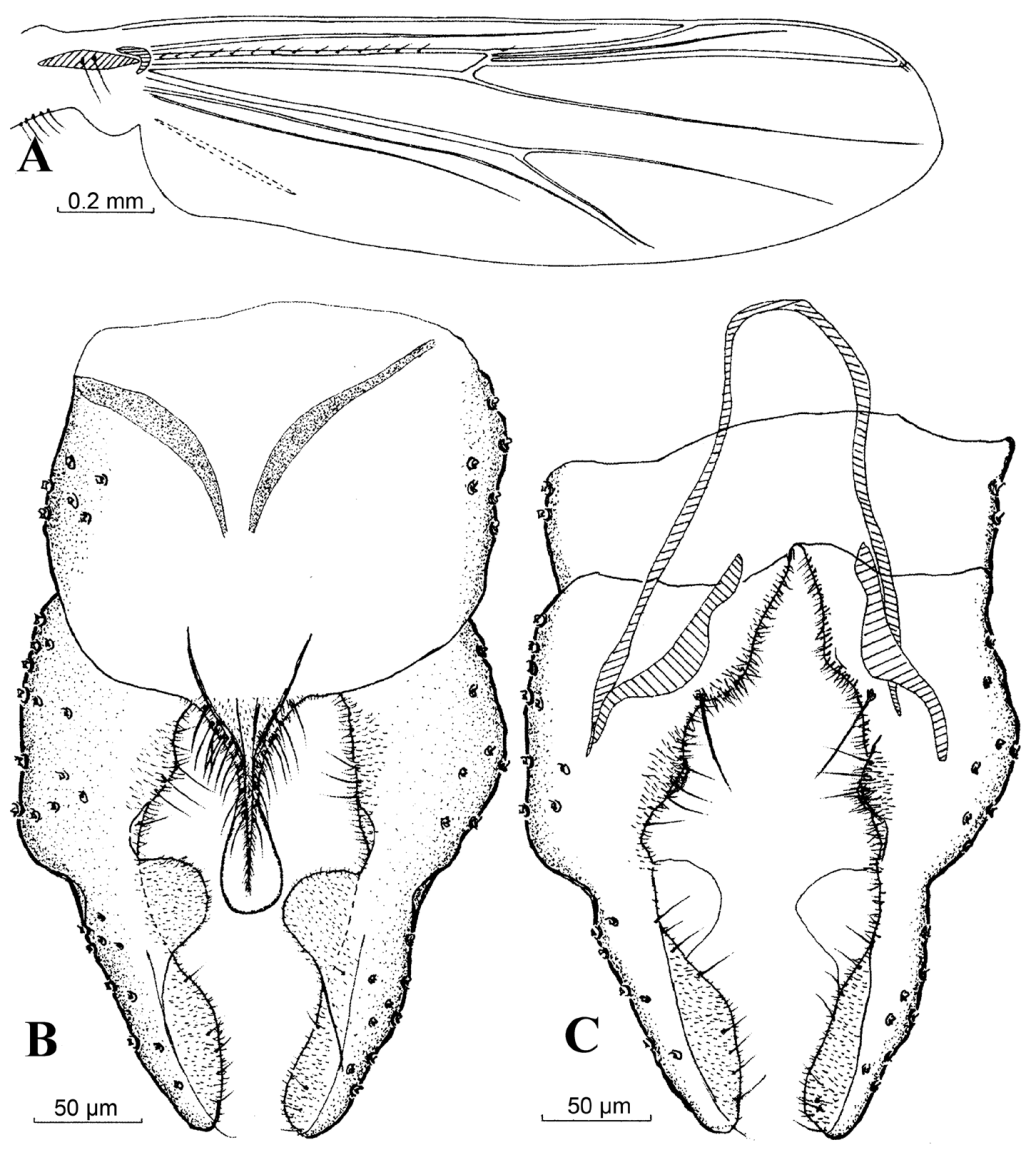
*Harnischia turgidula*. **A** Wing; Hypopygium: **B** (dorsal) **C** (ventral).

#### Distribution.

China (Hunan, Guangdong, Yunnan); Russian Far East.

### Harnischia sp.1

Taxon classificationAnimaliaDipteraChironomidae

Fig. 10

#### Diagnositic characters.

Pupal stage: sternite I with a pair of spinose anterolateral tubercles, dark and developed; tergites III-VI with narrow, posterior robust spines; hook row medially interrupted; segment II-IV with 2 L setae situated on tubercles; segment VIII without comb or spur.

#### Materials examined.

China: 2P, Jiangxi Province, Shangrao City, Poyang County, Poyang Lake 12.05.2015. W. Liu.

**Description. *Pupa*** [n = 2] Total length 5.8–6.0 mm. Exuviae brown.

**Figures 10.**
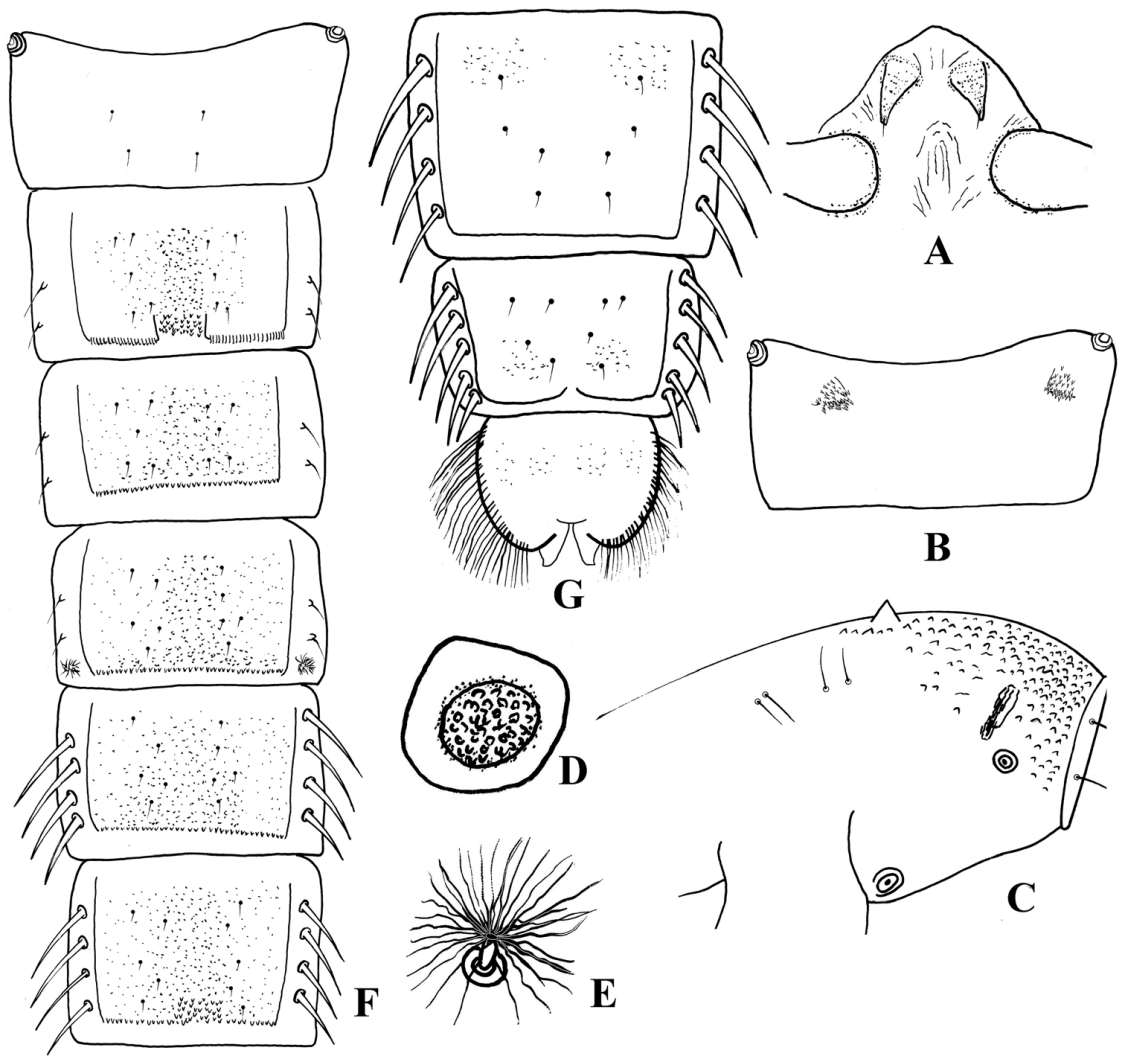
*Harnischia* sp.1. Pupae. **A** frontal apotome **B** sternite I **C** thorax, lateral view **D** basal ring **E** thorax horn **F** tergites I–VI **G** tergite VII–VIII and anal lobe.

*Cephalothorax* (Figs 10A, C–E). Frontal setae 25–30 µm long, fine, arising subapically from conical, 125–150 µm long cephalic tubercles. Thoracic horn plumose, with numerous fine branches (Fig. 10E); basal ring oval (Fig. 10D). Thorax granulose dorsally, more densely granulose anteriorly. Prealar tubercle low and rounded. Scutal tubercle prominent. Wing sheath without nose; pearl row absent. Two short precorneals, 2 short antepronotals and 4 dorsocentrals present. Lengths of dorsocentrals (µm): 100–105, 80–105, 75–90, 65–90.

*Abdomen* (Fig. 10B, F–G). Tergite I (Fig. 10F) bare; II with posteromedian group of small points; III-VI with narrow, transverse, posterior band of robust spines; VII with a pair of anterior patches fine shagreen; VIII with a pair of anterior and posterolateral patches of shagreen. Hook row widely interrupted medially. Conjunctives bare. Pedes spurii A present on segment IV; pedes spurii B present on segment I. Sternite I (10 B) with a pair of spinose anterolateral tubercles, dark and well developed. Segment VIII without posterolateral comb or spur. Segment II-IV with 2 strong L setae situated on tubercles; V-VII with 4 LS setae, VIII with 5 LS setae.

*Anal lobe* (Fig. 10G) 1.75–1.94 × as long as broad, with complete fringe of 68–72 lamelliform setae. Genital sac 300 µm long, extending beyond anal lobe.

#### Remarks.

This species can easily be separated from other known pupa of *Harnischia* species by the following characters: sternite I with a pair of spinose anterolateral tubercles, dark and well developed; tergites III-VI with narrow posterior robust spines; hook row medially interrupted; segment II-IV with 2 L setae situated on tubercles; anal lobe with complete fringe of 68–72 lamelliform setae.

### Key to male adults of known *Harnischia* species from China

**Table:** 

1	Gonostylus with apical tooth	**2**
–	Gonostylus without apical tooth	**3**
2	Inner margin of gonocoxite with obvious projection; gonostylus longer than gonocoxite, swollen and knife-like at the middle; apical teeth straight	***Harnischia curltriata* Wang**
–	Inner margin of gonocoxite with inconspicuous projection; gonostylus shorter than gonocoxite, not swollen at the middle; apical teeth hooked	***Harnischia angularis* Albu & Botnariuc**
3	Gonostylus with inner basal projection	**4**
–	Gonostylus without inner basal projection	**6**
4	Gonocoxite protrudes into vesicular-shape at base	***Harnischia turgidula* Wang & Zheng**
–	Gonocoxite slightly protruding, not bulb-like	**5**
5	Anal point taper-shaped, and the apex of anal point sharp ***Harnischia fuscimana* Kieffer**
–	Anal point parallel-sided, the apex of anal point broad and blunt	***Harnischia parallela* Yan & Wang, sp. n.**
6	Gonostylus with swollen in the apex	***Harnischia japonica* Hashimoto**
–	Gonostylus parallel-sided or moderately slender apically	**7**
7	Junction of the gonostylus and gonocoxites not as above; anal point swollen in the middle, with median ridges	***Harnischia curtilamellata* (Malloch)**
–	Junction of the gonostylus and gonocoxite shrunken; anal point not swollen in the middle	***Harnischia longispuria* Wang & Zheng**

### Key to pupae of known *Harnischia* species from China

**Table:** 

1	Sternite I with 1 pair of spinose anterolateral tubercles (Fig. 5B)	**2**
–	Sternite I with 2 pairs of spinose tubercules on each side, 1 anterolateral and 1 anteromedian (Figs 4B, 10B)	***Harnischia curtilamellata* (Malloch)**
2	Pairs of spinose anterolateral tubercles on sternite I brown; segment II-IV with 3 strong L setae	***Harnischia fuscimana* Kieffer**
–	Pairs of spinose anterolateral tubercles on sternite I dark and well-developed; segment II-IV with 2 L setae situated on tubercles	***Harnischia* sp.1**

## Supplementary Material

XML Treatment for Harnischia

XML Treatment for Harnischia angularis

XML Treatment for Harnischia cultriata

XML Treatment for Harnischia curtilamellata

XML Treatment for Harnischia fuscimana

XML Treatment for Harnischia japonica

XML Treatment for Harnischia longispuria

XML Treatment for Harnischia parallela

XML Treatment for Harnischia turgidula

XML Treatment for Harnischia sp.1
